# Orbital tuberculosis presenting as vision loss and headache: early management is paramount

**DOI:** 10.1186/s12348-024-00425-x

**Published:** 2024-10-14

**Authors:** Sandeep Pal, Narendra Patidar, Gunjan Tomar, Himanshu Gaikwad

**Affiliations:** 1 Department of Orbit and Oculoplasty, Sadguru Netra Chikitsalaya, Chitrakoot, India; 2 Consultant and Head of department, Orbit and Oculoplasty, Sadguru Netra Chikitsalaya, Jankikund, District Satna, Chitrakoot, Madhya Pradesh 210204 India

**Keywords:** Tuberculous, Orbital apex syndrome, Orbital TB

## Abstract

Orbital tuberculosis is a rare form of extra pulmonary TB and may arise either by hematogenous route or spread directly from the paranasal sinus. We herein report two cases of orbital TB with a vision threatening complication. Case-1 is a 31-year-old female with a headache, a diminution of vision in the right eye, and pain in ocular movement. On examination, there was no proptosis with RAPD present in right eye and tenderness on palpation. CEMRI revealed a diffuse infiltrating lesion at the orbital apex, suggesting of inflammatory pathology. Case-2 is a 40-year-old male with similar complaints in the left eye, CECT showed edema and swelling in the optic nerve and extraocular muscle of the left eye. A detailed investigation was done, and a diagnosis of orbital tuberculosis was made in both patients. They were started on ATT and oral steroids but lost follow-up initially and due to delayed treatment, it led to irreversible vision loss. A long-term follow-up showed resolution of ocular symptoms with occasional headaches.

## Introduction

Orbital TB is a rare entity of extrapulmonary TB; up to 60% of patients with extrapulmonary TB may not have been diagnosed with pulmonary TB [1]. Orbital apex syndrome refers to a constellation of symptoms and signs that result from the involvement of various structures in the region of the orbital apex by a disease process. The most common clinical features are vision loss and painful or limited extraocular movements [2]. The diagnosis of orbital TB is not straightforward sometimes due to difficulty in obtaining the tissue for biopsy, like in orbital apex lesion or optic nerve lesions. This has led to clinicians being reliant on clinico-radiological features, tuberculin skin tests and PCR.

Our report presents two cases of orbital tuberculosis with headaches and painful extraocular movement and highlights the vision-threatening complications due to their delayed presentation.

Permission from the patient to print identifiable photographs was obtained and archived. The case described in this report is compliant with the Declaration of Helsinki and Health Insurance Portability and Accountability Act regulations.

## Case presentation

### Case-1

**Fig. 1:**
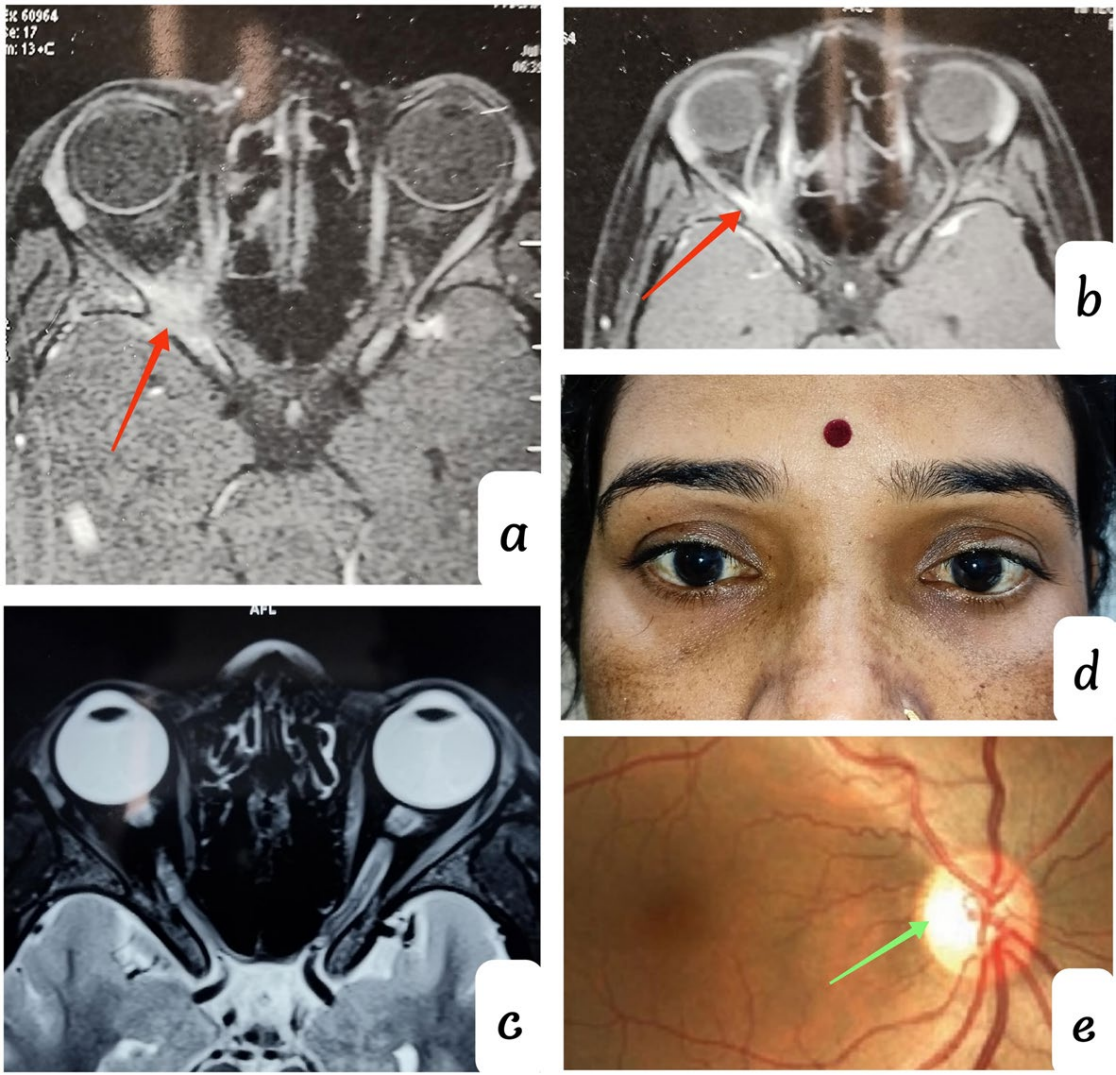
(**a** & **b**) CEMRI T1 axial section showing hyperintense lesion at right orbital apex on initial presentation (**c**) Resolved lesion at right orbital apex on 1-year follow-up (**d**) Clinical picture on 1-year follow-up (**e**) Right disc showing temporal pallor on 1-year follow-up

A 31-year-old female, presented with headache, diminution of vision in the right eye, and painful extraocular movement for 3-months. She initially consulted a neurologist for severe headache, followed by vision loss and drooping of the right eyelid, where she was prescribed opioid analgesics and anti-inflammatory drugs. CEMRI was advised, which revealed a hyper-intense lesion in the orbital apex, and the patient was referred to our OPD [Figure-1 a, b]. The drooping of the lid was resolved after the medication and the rest of the complaints were still persisting. The BCVA was 1/60 in the right eye and 6/6 in the left eye. On examination, there was no sign of proptosis, extraocular movements were painful, and without any restrictions. RAPD was present in the right eye and a fundus examination was clinically unremarkable. The systemic examination was clinically unremarkable, with no palpable lymph node. A comprehensive blood analysis (CBC, HIV, HbSAg, HCV, IgG4, C-ANCA, P-ANCA, RA factor, serum ACE, ANA, and HRCT chest was within normal limits (WNL).

The Montoux test was strongly positive with 30 × 25 mm induration after 72 h and.

QuantiFERON TB Gold tested positive. A clinical diagnosis of tuberculous orbital apex.

syndrome was made, and the patient was started on ATT with oral steroids. Patient didn’t take treatment and lost follow-up; one month later, she came with similar complaint and the planned treatment was initiated. She completed a 10-month ATT course (2 months of HRZE + 8 months of HR) with tapering oral corticosteroids as advised by the pulmonologist. At 1- year of follow-up, painful ocular movement was resolved completely, BCVA was similar 1/60 in the right eye with a persistent headache. A Fundus examination revealed right eye temporal disc pallor [Fig. 1-e]. A Repeat CEMRI orbit was advised, and the lesion at right orbital apex was completely resolved [Fig. 1-c].

### Case-2

**Fig. 2:**
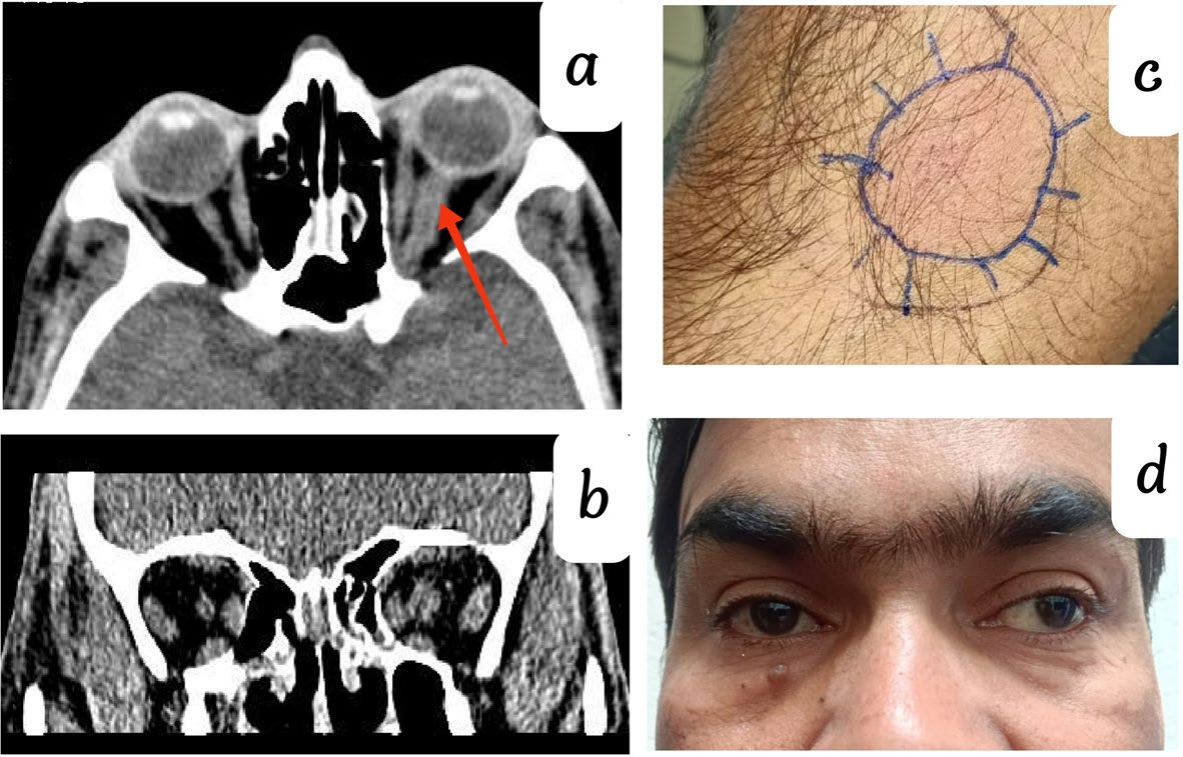
(**a** & **b**) CECT Orbit left eye showing swelling in optic nerve and extraocular muscle (**c**) Montoux test-24 × 26 mm (strongly positive) (**d**) Clinical picture on 1.8-year follow-up

A 40-year-old male with PL-positive on the left eye, presented with headache, painful extraocular movement, diminution of vision, and drooping of the left eyelid for 1 week. On examination, there was no sign of proptosis, with a mild limitation of extraocular movement in up gaze and down gaze. RAPD was present in the left eye with optic disc edema, systemic examination was clinically unremarkable. CECT orbit and blood investigations were advised but the patient denied it and lost follow-up. One week later, he came with a loss of vision in his left eye (PL-negative), CECT revealed swelling and edema in the left optic nerve and extraocular muscles suggestive of orbital inflammatory disease [Fig. 2-a, b]. He denied blood investigation, so IVMP pulse therapy was started immediately (5 cycles every 21 days). Drooping of the lid, painful and limited movement were improved without vision salvage (PL-negative) after complete pulse therapy. Two years after the completion of pulse therapy he came with a headache and painful ocular movement, showing signs of recurrence.

The detailed blood investigation revealed CBC, HIV, HbS, HCV, IgG4, C-ANCA, P-ANCA, RA factor, Serum ACE, ANA, QuantiFERON Tb Gold, and HRCT chest within normal limits (WNL). The Montoux test was strongly positive with 24 × 26 mm induration after 72 h [Fig. 2-c]. Thus, based on strong montoux positive, ruling out other orbital inflammatory diseases a clinical diagnosis of orbital tuberculosis was made, and he had completed 6 months of ATT (2 months of HRZE + 4 months of HR) with tapering oral corticosteroids as advised by the pulmonologist. After ATT completion, an 18-month follow-up revealed total resolution of ocular symptoms and no signs of recurrence [Fig. 2-d].

## Discussion

Hughes et al. described a case series of seven tuberculous OAS from 1975 to 2006. They quoted OAS as an optic neuropathy and/or ophthalmoplegia and sensory loss due to a lesion in the region of the orbital apex involving multiple cranial nerves. The mean age of the patients was 34.3-years, all presented with unilateral vision loss with pain on ocular movement, which is in accordance with our case. Two patients had previous close TB contact, which was not in our cases. All patients suffered constitutional symptoms with a mean duration of 21 days with headache being the most common (6/7 cases), flu-like symptoms (3/7 cases), and ATT with oral corticosteroids being started in all patients. Visual acuity was improved from Counting finger (6/7 cases) to 6/6 in all patients at a median follow-up of 30 weeks, which is in contrast to our case where visual acuity was not improve, which may be due patient’s poor compliance with starting the treatment [3]. Tanawade et al. reported a case of TB OAS with pulmonary involvement in a 16-year-old female with irreversible vision loss and severe limitations in ocular movement. She was started with high-dose steroids pulse therapy for 3 days along with ATT, improvement in extraocular movement with limited vision recovery was noted, which may be due to a delay in the presentation and recognition of optic nerve compression due to infiltrative lesion around optic nerve, which is in accord to our case, where similar infiltrative lesions was seen in orbital apex and optic nerve may be causing compression [4] Babu K et al. retrospectively analyzed six cases of orbital and adnexal TB from the south Indian population in 3-years. The varied presentation included TB dacryoadenitis (2), orbital periostitis (2), OTb osteomyelitis (1), and skin ulcer adnexal Tb (1), there were no cases of TB orbital apex syndrome reported [5].

In our report, it was found that Case-1 (TB-OAS) was initially presented as a severe headache followed by a diminution of vision, drooping of the lid and painful ocular movement without any restriction. CEMRI shows a lesion at the orbital apex, delay in presentation to an ophthalmologist may be the cause of irreversible vision loss in our case. After completion of ATT, painful ocular movement was resolved, and the patients has occasional headaches. Case-2 (orbital TB) also came with a similar presentation and mild movement restrictions. CECT shows swelling and inflammatory changes in the optic nerve and extraocular muscles. Strong positive montoux (24 × 26 mm) are unlikely to be due to previous BCG vaccination or exposure to environmental mycobacteria, as per Nayak S. et al. [6]. A Comparative analysis between tuberculin skin test (TST) and the QuantiFERON-TB Gold test by Shah Ira et al. mentioned that TST specificity increased to 63.2% by considering TSTs of 15 mm and above as positive [7]. A thorough literature search did not reveal such an atypical presentation of orbital tuberculosis. Poor compliance with the investigation, leading to a delay in treatment, may be the reason for irreversible vision loss.

Despite the paucibacillary nature of the disease, a biopsy is the mainstay for confirming orbital tuberculosis. Obtaining tissue from the site of infection in our cases carries the risk of morbidity and damage to vital structures near the orbital apex and optic nerve, so we relied on clinico-radiological analysis, the Montoux test and TB Gold for diagnosis. Although constitutional symptoms and the involvement of another cranial nerve that passes through the orbital apex were strong indicators of an alternative diagnosis, acute demyelinating optic neuropathy can be considered a differential diagnosis.

## Conclusion

This case emphasizes the significance of early diagnosis and treatment of orbital tuberculosis by ophthalmologists and neurologists in patients presenting with headaches and painful ocular movement to prevent the vision-threatening complications we noted in our report, where patients initially lost follow-up without taking treatment, making vision salvage difficult.

## Data Availability

No datasets were generated or analysed during the current study.

## Abbreviations

RE: Right Eye
LE: Left Eye
WNL: Within normal limit
IOP: Intraocular pressure
CEMRI: Contrast enhanced MRI
OPD: Outpatient department
TB OAS: Tuberculous orbital apex syndrome
ATT: Anti tubercular treatment
H: Isoniazid
R: Rifampicin
Z: Pyrazinamide
E: Ethambutol
